# Redistribution of charge in a 2D/1D BiOBr/Bi_2_O_2_S heterojunction for the photoelectrocatalytic oxidation of organic pollutants in water

**DOI:** 10.1039/d5ra03795f

**Published:** 2025-08-21

**Authors:** Kehinde D. Jayeola, Dimpo S. Sipuka, Tsholofelo I. Sebokolodi, Jonathan O. Babalola, Yumeng Zhao, Omotayo A. Arotiba

**Affiliations:** a Department of Chemical Sciences, University of Johannesburg Doornfontein Johannesburg South Africa oarotiba@uj.ac.za; b Centre for Nanomaterials Science Research, University of Johannesburg South Africa; c Department of Chemistry, University of Ibadan Ibadan Nigeria; d Bowen University Iwo Osun State Nigeria; e State Key Laboratory of Urban Water Resource and Environment, Harbin Institute of Technology Harbin 150090 China

## Abstract

This study presents the design of a 2D/1D BiOBr/Bi_2_O_2_S p–n heterojunction developed for the degradation of organic pollutants in water, addressing the issue of water contamination caused by pharmaceutical compounds. In an *in situ* hydrothermal synthesis method, the BiOBr nanosheets were synthesised on Bi_2_O_2_S nanorods in varying ratio to form a heterojunction that maximises charge separation and suppressed charge recombination. At an optimal 20% Bi_2_O_2_S ratio, BiOBr/Bi_2_O_2_S heterojunction achieved 88% degradation efficiency of ciprofloxacin and TOC removal of 60%, when compared with the pristine BiOBr and Bi_2_O_2_S. The wider application of the photoanode was investigated by degrading other pollutants like tetracycline and sulfamethoxazole. Comprehensive structural, optical, and electrochemical analyses confirmed the increased surface area and active sites, enhanced light properties and better charged separation. The radical trapping studies identified the hydroxyl radical as a primary contributor to the degradation process, indication the p–n heterojunction facilitated by the formation of a space charge region. This study establishes the BiOBr/Bi_2_O_2_S as an effective photoanode for PEC water treatment and provides a promising approach to mitigate organic pollutant detection in water.

## Abstract 2

This study presents the design of a 2D/1D BiOBr/Bi_2_O_2_S p–n heterojunction developed for the degradation of organic pollutants in water, addressing the issue of water contamination caused by pharmaceutical compounds.

## Graphical abstract

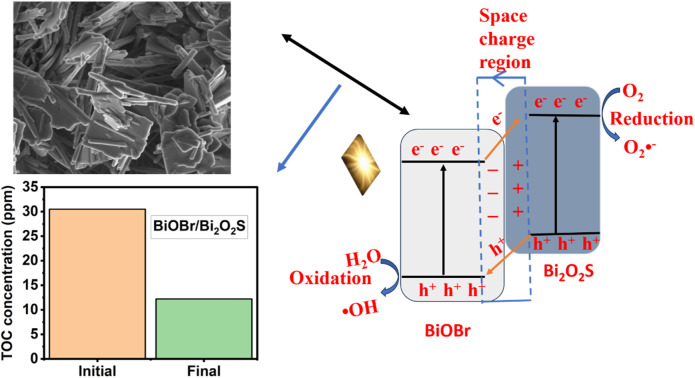

## 1. Introduction

The contamination of water by persistent organic pollutants including pharmaceuticals poses a severe threat to the environment and human health.^1,2^ Among several water treatment technologies, photoelectrochemical oxidation (PEC) has emerged to be a promising solution due to its ability to drive a complete mineralisation of pollutants under mild conditions with less production of toxic intermediates and by products.^3–5^ PEC system harnesses light and electrical energy to generate powerful oxidants for the degradation of pollutants in aqueous solution. In a typical PEC set up, the semiconductor photoanode absorbs light and generate charge carriers, then the photogenerated holes will migrate to the electrode surface, where they react with water molecules or hydroxyl ions to generate reactive oxygen species (ROS) like hydroxyl radicals, or even degrade pollutant directly.^6,7^ These ROS oxidise and break down organic pollutants in water into smaller molecules like CO_2_ and H_2_O. Meanwhile, the photogenerated electrons migrates through the applied voltage/current to the cathode, where it indirectly takes part in the oxidation of the pollutant by reducing absorbed oxygen and converting it into superoxide anion radical.^8^

Moreover, recent advancements in PEC degradation process have been centred on the use of visible light active materials that can be activated with sunlight energy and has better stability for applicability and scalability purposes.^9^ Therefore, there has been a rise in exploring the properties of the two-dimension morphology in heterojunction formation.^10^ 2D semiconductors have gained attraction due to some unique properties. They possess high surface-area to volume ratio that can enhance light absorption and facilitates charge transfer, as thinner nanosheets have been reported to require shorter migration distance for charge carrier movement.^11–13^ An example is BiOBr nanosheet, which has layered structure, suitable band gap, and high chemical stability.^14^ In addition, BiOBr is a p-type semiconductor and possesses band gap energy ranging from 2.6–2.9 eV. However, BiOBr has drawbacks such as limited light absorption in the visible spectrum and relatively low charge mobility which affects its photoelectrocatalytic efficiency.^15^ An attempt to solve these challenges led to the formation of BiOBr based heterojunction. Semiconductor heterojunctions such as BiOBr/TiO_2_,^16^ g-C_3_N_4_/BiOBr,^17^ WO_3_/BiOBr,^18^ BiOBr/BiOI,^19^ BiOBr/Bi_2_WO_6_,^20^ and so on have been explored for photocatalysis and PEC water splitting, but the application of BiOBr based heterojunction is still limited for PEC water treatment.^21,22^

On the other hand, Bi_2_O_2_S is also a bismuth-based semiconductor with narrow band gap between 1.3–1.5 eV that facilitates absorption in the visible light region.^23,24^ It consists of a layered structure that promotes strong in-plane covalent bond and weak interlayer van der Waals interaction that helps to facilitate charge transport. Bi_2_O_2_S exhibits high electron mobility, chemical stability and conductive active sites that assist in the effective degradation of pollutants.^25^ Therefore, combining BiOBr and Bi_2_O_2_S semiconductors to form a hetero-structured photoanode will offer a better PEC water treatment system. The heterojunction formation BiOBr and Bi_2_O_2_S semiconductors can create a favourable band alignment, which can potentially lead to a p–n junction configuration that can improve photoelectrochemical degradation efficiency.

In a p–n heterojunction, electron moves from the n-type semiconductor (excess electron) to the p-type semiconductor (excess holes) creating a negative charge on the surface of the p-type semiconductor and a positive charge on the n-type semiconductor, thus a depletion region at the interface. This electron diffusion continues until the Fermi level reaches equilibrium resulting in a space charge region at the p–n junction. When light is irradiated, both semiconductors will generate electrons and holes; however, the space charge region (built in electric field) will separate this by pushing the electrons to the n-type side and the holes to the p-type side. This separation of charges facilitates effective photoelectrochemical degradation process of pollutant^19,26^

To interrogate the possible enhancement of PEC activity of the proposed BiOBr/Bi_2_O_2_S p–n heterojunction, we used ciprofloxacin, a commonly used fluoroquinolone antibiotics known for its effectiveness in treating various type of bacterial infections as a model pollutant.^27^ Despite its efficacy as a drug, ciprofloxacin has become a significant environmental pollutant because of its persistence in water bodies, where it becomes resistant and accumulates over time. This persistence poses ecological risks including the development of antibiotic-resistant bacteria and adverse effect on aquatic bodies and humans' health.^28^ Therefore, there is a need to generate efficient photoanode to remove ciprofloxacin from water. With the design of BiOBr based photoanode such as BiOBr/Bi_2_O_2_S, we aim to explore the complementary electronic properties of each semiconductor for efficient charge transfer pathways leading to improved PEC performance. The synergistic combination of these semiconductors can be optimised to achieve better stability, light absorption and degradation efficiency.

Thus, this study is on the synthesis, structural properties, morphology, optical properties and charge transfer mechanism of 2D/1D BiOBr/Bi_2_O_2_S heterojunction photoanode for the PEC degradation of emerging contaminants in water. The choice of the pristine BiOBr is motivated by its inherent stability, layered structure and moderate band gap. In addition, BiOBr exhibits band gaps between 2.5–2.8 eV which allows for better visible light absorption and strong oxidation potential when compared with BiOCl^29^ and BiOI.^30^ Although, BiOI possess band gaps between 1.7–1.9 eV with strong visible light absorption abilities, it has weak redox capabilities. While Bi_2_O_2_S was selected for its band structure and visible light absorption properties. To the best of our knowledge, this is the first study to report the fabrication, characterisation, and application of 2D/1D BiOBr/Bi_2_O_2_S heterojunction photoanode for PEC water treatment thus informing future efforts in designing heterojunction based photoanodes. Table 1 shows a comparative study with reported materials in literature to highlight that BiOBr/Bi_2_O_2_S is a promising material for water treatment.

**Table 1:** Reported materials for the photodegradation of organic pollutants

Semiconductors	Target analyte	Degradation efficiency, time	Reaction kinetics	Electrolyte	Ref.
FTO/NiSe_2_/BiVO_4_	Ciprofloxacin	76%, 180 min	0.0065 min^−1^	Na_2_SO_4_	31
Zr:BiVO_4_@Bi_2_S_3_-CoS	Tetracycline hydrochloride	94%, 60 min	0.099 min^−1^	Na_2_S/Na_2_SO_4_	32
FTO/BiVO_4_/BiOI	Ciprofloxacin	62%, 120 min	0.007 min^−1^	Na_2_SO_4_	33
Bi_2_WO_6_-CNP-TiO_2_	Paracetamol	84%, 180 min	0.009 min^−1^	Na_2_SO_4_	34
Fe/HNHC-PMS system	Bisphenol A	92.5%, 30 min	0.068 min^−1^	Not specified	35
CoNC/NHCNTs (PMS/photocatalysis)	Tetracycline	Close to100%, 30 min	Not specified	—	36
WO_3_/BiVO_4_	Diclofenac	75–80%	Not specified	Na_2_SO_4_	37
WO_3_–g-C_3_N_4_/grafoil	Ciprofloxacin	75%, 75 min	0.004 min^−1^	Na_2_SO_4_	38
BiOBr/BiOCOOH (photocatalysis)	Levofloxacin	90.1%, 40 min	0.055 min^−1^	—	39
MgIn_2_S_4_/CeO_2_ (photocatalysis)	Tetracycline	86%, 120 min	0.027 min^−1^	—	40
rGO/BiOBr/TiO_2_	*p*-Chloronitrobenzene	86.8%, 360 min	0.005 min^−1^	—	41
Pt@CeO_2_@MoS_2_ (PMS)	Carbamazepine	85%, 30 min	0.132 min^−1^	—	42
BiOBr@ZnIn_2_S_4_ (phocatalysis)	Methyl orange	95%, 40 min	0.07 min^−1^	—	43
CuO/BiOCl	Aflatoxin B1	81.3%, 180 min	Not specified	—	44
Bi_2_O_2_S/NiTiO_3_	Sulfamethoxazole	80%, 180 min	0.008 min^−1^	Na_2_SO_4_	45
BiOBr/Bi_2_O_2_S	Ciprofloxacin	88%, 180 min	0.0127 min^−1^	Na_2_SO_4_	This work

## 2. Materials and METHODS

### 2.1. Materials used

Thiourea (CH_4_N_2_S), polyvinylidene fluoride (PVDF), lithium hydroxide monohydrate (LiOH H_2_O), bismuth nitrate pentahydrate (Bi(NO_3_)_3_·5H_2_O), potassium bromide (KBr), acrylamide, water, potassium ferrocyanide (K_4_[Fe(CN)_6_]), sodium sulphate (Na_2_SO_4_), *tert*-butanol, sodium ethylenediaminetetraacetate (EDTA), fluorine doped tin oxide glass, and *N*-methyl-2-pyrrolidone (NMP).

### 2.2. Photoanode preparation

#### 2.2.1. Synthesis of BiOBr

The BiOBr nanosheets were synthesised using an hydrothermal method that was reported by Li *et al.*,^46^ with slight modification. 0.002 moles of (Bi(NO_3_)_3_·5H_2_O) and 0.0021 mol of KBr is added into 50 mL of deionised water and sonicated for 30 min. The obtained mixture was then heated at 180 °C for 24 hours. The product was then cooled, washed with ethanol and deionised water several times, followed by drying for 8 hours.

#### 2.2.2. Synthesis of Bi_2_O_2_S

The hydrothermal method of synthesis that was previously reported^23^ was employed with slight modification for the preparation of Bi_2_O_2_S nanorods. 0.004 moles of (Bi(NO_3_)_3_·5H_2_O) and 0.002 moles of CH_4_N_2_S were dissolved in 40 mL of deionized water and sonicated for 20 min. then 0.28 moles of LiOH H_2_O were added and further sonicated for 60 min until a uniform reddish solution is formed. The solution was then transferred to crucible and placed in the furnace for 72 hours at 200 °C. After which the obtained product was washed thoroughly with ethanol and deionised severally and dried at 60 °C for 8 hours.

#### 2.2.3. Synthesis of BiOBr/Bi_2_O_2_S

For the BiOBr/Bi_2_O_2_S heterojunction, four mole ratios (5%, 10%, 20%, and 30%) of Bi_2_O_2_S to BiOBr were synthesised *via in situ* hydrothermal method. The respective masses of the synthesised Bi_2_O_2_S were dispersed in deionised water inside four different beakers. Each beaker containing 5%, 10%, 20%, and 30% of Bi_2_O_2_S were sonicated for 30 min, followed by the synthesis of BiOBr as reported in Section 2.2.1.

#### 2.2.4. Fabrication of photoanode

Drop-coating method was employed in the fabrication of BiOBr, Bi_2_O_2_S, and BiOBr/Bi_2_O_2_S photoanodes, on a fluorine doped tin oxide (FTO) glass substrate. A slurry paste formed from 50 mg of synthesised nanoparticles, 90 μL of *N*-methyl-2-pyrrolidone, and 5 mg of polyvinylidene fluoride was coated on the FTO glass using a geometrical area of 1.7 cm by 1.7 cm. The coated FTO was then dried in the oven at 60 °C for 2 h.

### 2.3. Characterisation

The crystallinity and composition of the synthesised materials were performed using X-ray diffraction (XRD) (Cu Kα radiation, Rigaku Ultima IV, Japan). The elemental composition and surface valence were analysed using X-ray photoelectron spectroscopy (XPS) and field-emission scanning electron microscope and Energy dispersive X-ray (FE-SEM-EDX) (JEOL JSM-7500F, Japan). The optical properties were examined using ultraviolet-visible diffuse reflectance spectrophotometry (UV-DRS) (Cary 60 UV-Vis, Malaysia). The recombination rate of photogenerated charge carriers was investigated using photoluminescence spectroscopy (PL) (F-186 2710, HITACHI, Japan). Surface morphologies of the semiconductors were investigated using transmission electron microscopy (TEM) (JOEL, Germany) and field-emission scanning electron microscope (FE-SEM) (JEOL JSM-7500F, Japan). The surface charge of the fabricated semiconductors was measured using the Zetasizer instrument (ZEECOM ZC 300, Japan). The degradation efficiency was monitored using UV-Vis spectroscopy (Cary 60 UV-Vis, Malaysia). The degradation pathway was monitored using ultra-performance liquid chromatography-mass spectrometry (UPLC-MS) (WATERs, USA). The surface area, pore size and pore volume were investigated using the Brunauer–Emmett–Teller (BET) analysis (Micrometrics Corporation ASAP 2020 V4.00). The effect of temperature on the synthesis semiconductors were investigated using the thermogravimetric analysis (TGA Q500, TA Instruments, USA).

The photodegradation measurement and degradation procedure are presented in Section S1 of the SI section.

## 3. Results and discussions

### 3.1. Structural, surface, and morphology studies

**Fig. 1:**
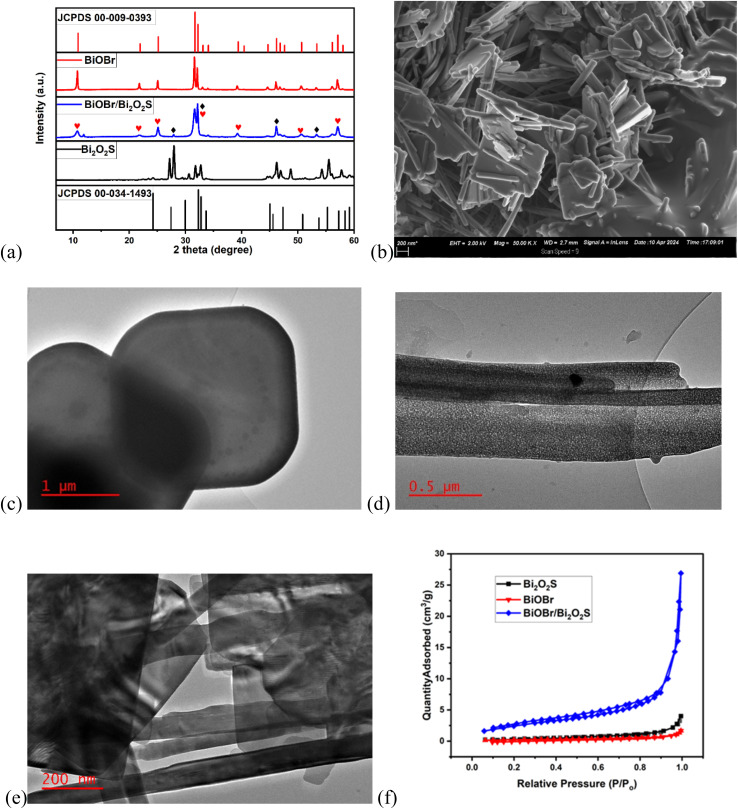
(a) XRD pattern of BiOBr, Bi_2_O_2_S and BiOBr/Bi_2_O_2_S (b) FESEM micrograph of BiOBr/Bi_2_O_2_S. TEM micrograph (c) BiOBr, (d) Bi_2_O_2_S, (e) BiOBr/Bi_2_O_2_S. (f) BET Isotherm curve of BiOBr, Bi_2_O_2_S and BiOBr/Bi_2_O_2_S.

Fig. 1a shows XRD diffraction pattern of the synthesised BiOBr, Bi_2_O_2_S, and BiOBr/Bi_2_O_2_S. The major peaks of BiOBr at the 2 theta 10.77°, 21.77°, 25.08°, 31.55°, 32.14°, 39.20°, 46.14°, 50.66°, & 57.07 are indexed to the *hkl* (001), (002), (101), (102), (110), (112), (200), (104) and (212) planes of the BiOBr tetragonal phase (JCPDS #00-009-0393).^47^ On the other hand, the peaks of Bi_2_O_2_S at 2 theta 27.18°, 29.53°, 31.81°, 32.78°, 46.2°, 48.80°, 54.21°, 55.62°, & 57.83° are indexed to the *hkl* (120), (040), (130), (101), (060), (002), (112), (221), and (161) planes of Bi_2_O_2_S orthorhombic phase (JCPDS #01-085-0451).^48^ The diffraction pattern of BiOBr/Bi_2_O_2_S show peaks at 2 theta 10.77°, 21.77°, 25.08°, 31.70°, 32.14°, 39.20°, 46.2°, 56.13°, & 57.14° corresponds to the main peaks of BiOBr and Bi_2_O_2_S. Peaks overlaps which led to a peak broadening are observed at 2 theta 31.70° and 32.14° which corresponds to the *hkl* (130) & (101) and (102) & (110) plane of Bi_2_O_2_S and BiOBr respectively, thus confirming the formation of heterojunction.

The FESEM micrograph of BiOBr (Fig. S1) shows a layered sheet-like structure with a two-dimension (2D) morphology. The nanosheet appeared to be thin, flat and well distributed, forming an interconnected structure. The FESEM micrograph of Bi_2_O_2_S (Fig. S2) reveals a rod-like, one-dimensional (1D) morphology, that displayed a high aspect ratio with elongated structures which are uniformly distributed and interconnected. FESEM micrograph of BiOBr/Bi_2_O_2_S (Fig. 1b) shows the combination of the rod-like and sheet-like structure of Bi_2_O_2_S and BiOBr respectively. The rods appeared to be intertwined within the flatter nanosheets, leading to increased surface area and active sites for photoelectrochemical degradation. Furthermore, the TEM micrograph BiOBr (Fig. 1c) confirms the interconnected sheet-like structure. Also, the TEM micrograph of Bi_2_O_2_S (Fig. 1d) confirms the elongated rod-like morphology, with rough edges and texture. And, the TEM micrograph of BiOBr/Bi_2_O_2_S (Fig. 1e) shows the intertwined and interconnected pattern of BiOBr nanosheet and Bi_2_O_2_S nanorod. The combination of nanosheet and nanorod structure in the heterojunction photoanode led to an increase in degradation efficiency due to improved charge separation and increased light absorption properties of the unique morphologies.

The N_2_ adsorption–desorption isotherm curve of BiOBr, Bi_2_O_2_S, & BiOBr/Bi_2_O_2_S (Fig. 1f) shows hysteresis loops that are often observed in Type IV isotherms. This type of isotherm is characteristics of mesoporous materials. The Brunauer–Emmett–Teller (BET) and Barrett–Joyner–Halenda (BJH) analyses were used to estimate the surface area and pore volume respectively, of BiOBr, Bi_2_O_2_S, & BiOBr/Bi_2_O_2_S. The surface area of 0.958 m^2^ g^−1^, 1.581 m^2^ g^−1^, & 9.4045 m^2^ g^−1^ were calculated for BiOBr, Bi_2_O_2_S, and BiOBr/Bi_2_O_2_S respectively, with the corresponding pore volume of 0.0025 cm^3^ g^−1^, 0.0062 cm^3^ g^−1^, & 0.4160 cm^3^ g^−1^. Therefore, the heterojunction formation led to increased surface area and pore volume, which are useful for efficient light harvesting.^49^

The FTIR spectra of BiOBr, Bi_2_O_2_S, and BiOBr/Bi_2_O_2_S (Fig. S3) reveal the functional groups that are present. For BiOBr spectra, the peak at 3458 cm^−1^ and 1639 cm^−1^ can be ascribed to the O–H stretching and bending vibrations due to absorbed water. The peaks at 2033 cm^−1^ and 1415 cm^−1^ can be attributed to C–O stretching and bending vibrations due to absorbed atmospheric CO_2_. These peaks are mostly associated with samples prepared using KBr. While, the peak positioned at 1275 cm^−1^ and 523 cm^−1^ are attributed to stretching vibration of Bi–O bonds associated with halogenated bismuth compounds.^50^ The absorption bands of Bi_2_O_2_S at 1420 cm^−1^ corresponds to the S–O bending vibration. And, the peaks at 867 cm^−1^ & 484 cm^−1^ correspond to Bi–O stretching and bending vibration, respectively.^23^ All these characteristic peaks of BiOBr and Bi_2_O_2_S were observed in the spectra of BiOBr/Bi_2_O_2_S with an appearance of a new peak at 607 cm^−1^. The appearance of this peak suggests a possible modification around the Bi–O and S–O bonds due to heterojunction formation.^51^

### 3.2. Elemental composition

**Fig. 2:**
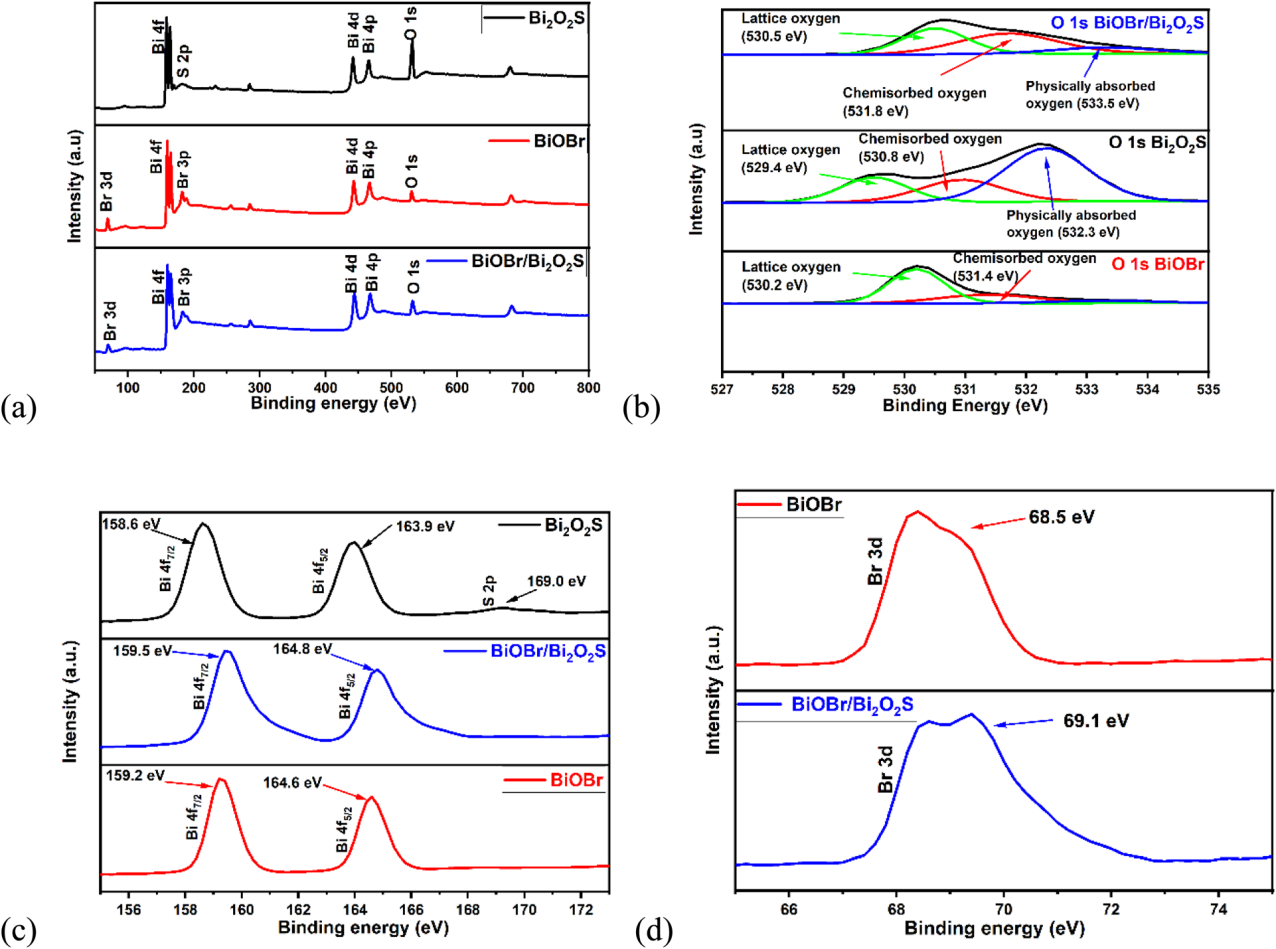
(a) XPS survey scan. XPS spectra of (b) O 1s, (c) Bi 4f and S 2p, of BiOBr, Bi_2_O_2_S and BiOBr/Bi_2_O_2_S. (d) Br 3d of BiOBr and BiOBr/Bi_2_O_2_S

The XPS survey scans of BiOBr, Bi_2_O_2_S and BiOBr/Bi_2_O_2_S (Fig. 2a) reveal the presence of the bismuth, sulphur, oxygen, and bromine. The O 1s spectra (Fig. 2b) show peaks with binding energy of 529. 4 eV, 530.8 eV & 532.3 eV for Bi_2_O_2_S which correspond to the lattice (Bi–O), chemisorbed, and physically absorbed oxygen, while the peaks at 530.2 eV, and 531.4 eV observed for BiOBr corresponds to the lattice (Bi–O) and chemisorbed oxygen. The positive shift in binding energy of 530.5 eV, 531.8 eV, and 533.5 eV observed for BiOBr/Bi_2_O_2_S for the lattice, chemisorbed, and physically absorbed oxygen is due to the increased interaction of the metal–oxygen bonds in BiOBr and Bi_2_O_2_S, resulting from heterojunction formation. Additionally, the Bi 4f spectra (Fig. 2c) reveals the Bi 4f peaks at 158.6 eV and 163.9 eV for Bi_2_O_2_S, and at 159.2 eV and 164.6 eV for BiOBr. These peaks shifted to 159.5 eV and 164.8 eV in BiOBr/Bi_2_O_2_S corresponds to Bi 4f_7/2_ and Bi 4f_5/2_ respectively. The S 2p orbital peak was observed at 169 eV for Bi_2_O_2_S, although it was absent in the BiOBr/Bi_2_O_2_S spectra. This could be due to the low percentage of sulphur in the composite thus falling below the detection limit of XPS. Moreover, the FESEM-EDX spectra (Fig. S4) confirmed the presence of bismuth, bromide, oxygen, as well as sulphur in the BiOBr/Bi_2_O_2_S heterojunction, where sulphur is detected in a low percentage of 0.3%. Furthermore, the Br 3d spectra shows peak at 68.5 eV of BiOBr, which shifted to 69.1 eV in BiOBr/Bi_2_O_2_S. These shifts to a higher binding energy of the orbitals of both BiOBr and Bi_2_O_2_S indicate a change in electron density which causes a redistribution of charges across the interface of the semiconductor.^52^ This could suggest that the electronic interaction between BiOBr and Bi_2_O_2_S facilitated charge separation to promote charge mobility.

### 3.3. Photo(electro)chemical properties

**Fig. 3:**
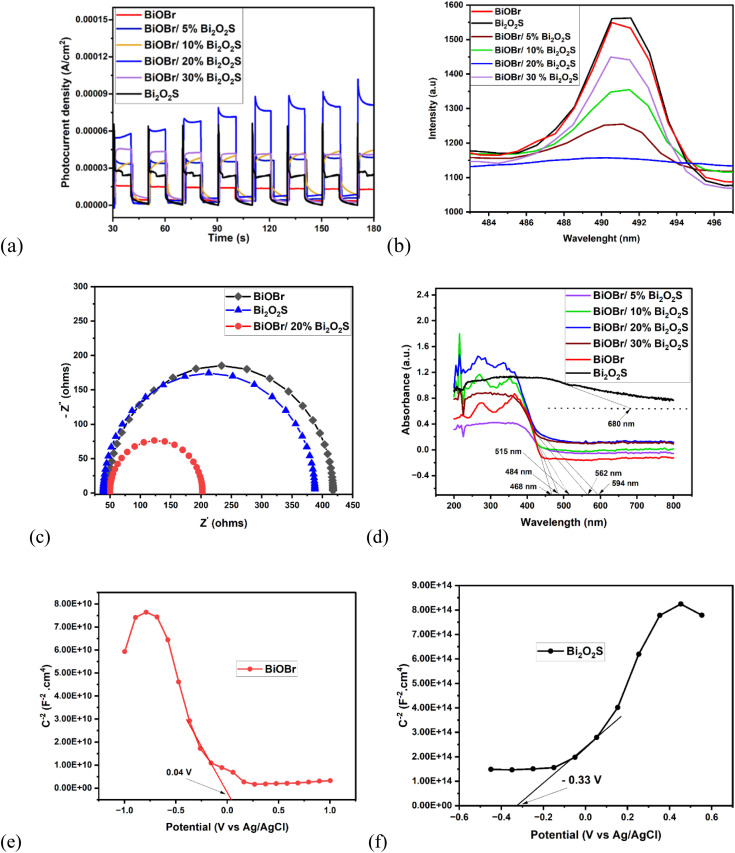
(a)Transient photocurrent response plot, (b) photoluminescence spectra, of BiOBr, Bi_2_O_2_S, BiOBr/5% Bi_2_O_2_S, BiOBr/10% Bi_2_O_2_S, BiOBr/20% Bi_2_O_2_S, & BiOBr/30% Bi_2_O_2_S. (c) EIS Nyquist plot BiOBr, Bi_2_O_2_S, & BiOBr/Bi_2_O_2_S, (d) UV-Vis DRS spectra of BiOBr, Bi_2_O_2_S, BiOBr/5% Bi_2_O_2_S, BiOBr/10% Bi_2_O_2_S, BiOBr/20% Bi_2_O_2_S, & BiOBr/30% Bi_2_O_2_S. Mott Schottky curve of (e) BiOBr, (f) Bi_2_O_2_S

As shown in Fig. 3a, the photocurrent densities of BiOBr, Bi_2_O_2_S, BiOBr/5% Bi_2_O_2_S, BiOBr/10% Bi_2_O_2_S, BiOBr/20% Bi_2_O_2_S, & BiOBr/30% Bi_2_O_2_S photoanodes were deduced as 0.015 mA cm^−2^, 0.065 mA cm^−2^, 0.030 mA cm^−2^, 0.037 mA cm^−2^, 0.212 mA cm^−2^, and 0.041 mA cm^−2^ respectively. Amongst the four different mole ratios, the BiOBr/20% Bi_2_O_2_S gave the highest densities which is 14 times greater than the photocurrent density of BiOBr and 3 times greater than that of Bi_2_O_2_S. This suggests that the BiOBr/20% Bi_2_O_2_S is more capable of light harvesting, efficient charge separation and better degradation efficiency.

Likewise, the photoluminescence spectra (Fig. 3b) reveal that the rate of recombination of photogenerated holes and electrons were more suppressed in the BiOBr/20% Bi_2_O_2_S heterojunction when compared to BiOBr/5% Bi_2_O_2_S, BiOBr/10% Bi_2_O_2_S, and BiOBr/30% Bi_2_O_2_S. A lower PL intensity suggests a lower or suppressed recombination rate of charge carriers.^23^ Moreover, the charge transfer resistance of the photoanodes using the EIS Nyquist plot (Fig. 3c) were found to be 372 Ω, 353 Ω, and 153 Ω for BiOBr, Bi_2_O_2_S, and BiOBr/20% Bi_2_O_2_S respectively. This suggests that BiOBr/Bi_2_O_2_S exhibits improved charge transfer efficiency with lower resistance of electron mobility which contributes to its superior performance in the PEC degradation process.

Also, the light absorption properties of the photoanodes were investigated using the UV-Vis DRS. As displayed in Fig. 3d, the pristine BiOBr has an absorption edge of 468 nm, indicating a wider band gap which limits its light absorption to the UV region, while Bi_2_O_2_S has an absorption edge of 680 nm, indicating a narrow band gap which allows for absorption in a broader spectrum of the visible light. BiOBr/5% Bi_2_O_2_S, BiOBr/10% Bi_2_O_2_S, BiOBr/15% Bi_2_O_2_S and BiOBr/30% Bi_2_O_2_S gave absorption edge of 484 nm, 515 nm, 562 nm, & 594 nm respectively. This implies that the increase in the mole ratio of Bi_2_O_2_S to BiOBr in the heterojunction lead to increase in narrowing of BiOBr/Bi_2_O_2_S heterojunction band gap.

### 3.4. Band energies calculation

Using eqn (1), the band gap energy of BiOBr (Fig. S5) and Bi_2_O_2_S (Fig. S6) were deduced to be 2.61 eV and 1.32 eV respectively.

1 *εhν* = *C*(*hν* − *E*_g_)^*n*^

*C* is the constant, *n* is determined by the type of transition, *E*_g_ is the band gap energy, and *hν* is the incident photon energy.

Also, the conduction and valence band energies were calculated based on vacuum scale and electronegativity using eqn (2) and (3).

2 *E*_CB_ = *X* − *E*_C_ − 0.5 *E*_g_

3 *E*_CB_ = *E*_VB_ − *E*_g_

*E*_VB_ = valence band, *E*_CB_ = conduction band, *E*_C_ = energy of free electrons (4.5 eV), *X* = Mulliken electronegativity, and *E*_g_ = band gap energy.

The *X* values for BiOBr and Bi_2_O_2_S were calculated to be 6.17 eV (ref. 53) and 4.81 eV (ref. 48) respectively. Therefore, the *E*_CB(NHE)_ of BiOBr and Bi_2_O_2_S are +0.37 eV and −0.34 eV with the corresponding *E*_VB(NHE)_ of 2.96 eV and 0.94 eV, respectively.

The negative and positive slope observed in the Mott Schottky curve of BiOBr (Fig. 3e) and Bi_2_O_2_S (Fig. 3f) confirmed that BiOBr is a p-type semiconductor, while Bi_2_O_2_S is a n-type semiconductor. Moreover, the flat band potentials (*E*_f_) were deduced with respect to Ag/AgCl electrode. The *E*_f_ values of +0.04 and −0.33 V were obtained for BiOBr and Bi_2_O_2_S respectively. From eqn (4), the *E*_f_ was calculated with respect to the normal hydrogen electrode. Hence, the *E*_f(NHE)_ approximately is 0.23 eV and −0.13 eV which is related to the Fermi level position with respect to the conduction band of BiOBr and Bi_2_O_2_S respectively.

4 *E*_(NHE)_ = *E*_(Ag/AgCl)_ + 0.194

In addition, the XPS valence spectra was used to calculate the *E*_VB(XPS)_ which is with respect to the Fermi level. The valence band maxima (VBM) of 2.57 eV and 0.54 eV were deduced for BiOBr (Fig. S7) and Bi_2_O_2_S (Fig. S8) respectively. The relationship between the *E*_VB(NHE)_ and *E*_VB(XPS)_ is shown in eqn (5).^54^

5 *E*_VB,NHE_ = *ϕ* + *E*_VB,XPS_ − 4.44

where, the work function (*ϕ*) of the XPS instrument is given as 4.8 eV.

Therefore, the expected *E*_VB(NHE)_ for BiOBr and Bi_2_O_2_S are 2.93 eV and 0.90 eV respectively. These values agree with the calculated *E*_VB(NHE)_ with a difference of less than 0.1.

### 3.5. Photoelectrochemical degradation

#### 3.5.1. Effect of heterojunction, pH, analytes and TOC studies

**Fig. 4:**
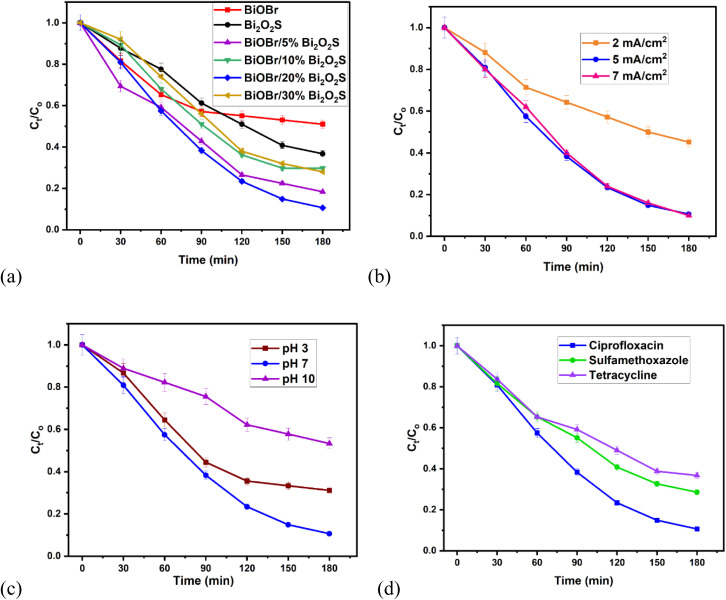
The PEC degradation efficiency plot showing (a) effect of heterojunction, (b) effect of current density, (c) effect of pH, (d) different analyte study (experimental conditions: ciprofloxacin concentration = 5 mg L^−1^ in 0.1 M Na_2_SO_4_, current density = 5 mA cm^−2^)

Ciprofloxacin was selected as the targeted organic pollutant to investigate the photoelectrocatalytic efficiency of the fabricated BiOBr/Bi_2_O_2_S heterojunction. BiOBr/20% Bi_2_O_2_S gave the highest degradation efficiency of 88% of CIP after 180 min, when compared to BiOBr, Bi_2_O_2_S, BiOBr/5% Bi_2_O_2_S, BiOBr/10% Bi_2_O_2_S, & BiOBr/30% Bi_2_O_2_S which gave 49%, 63%, 82%, 70% and 72% respectively (Fig. 4a). This shows that the BiOBr/20% Bi_2_O_2_S heterojunction demonstrated enhanced degradation performance over the pristine BiOBr and Bi_2_O_2_S. Therefore, the 20% Bi_2_O_2_S was used as the optimised heterojunction ratio. In addition, the data obtained were fitted linearly using the pseudo first order reaction model to obtain the rate of rection. As displayed in Fig. S9, the *k* values obtained for BiOBr, Bi_2_O_2_ & BiOBr/20% Bi_2_O_2_S are 0.0036 min^−1^, 0.0059 min^−1^, and 0.0131 min^−1^ respectively. This shows that the formation of heterojunction led to a faster rate of reaction.

The effect of current density on the degradation efficiency of BiOBr/20% Bi_2_O_2_S was also investigated. The current density varied from 2 mA cm^−2^ to 7 mA cm^−2^ (Fig. 4b), at 2 mA cm^−2^, the lowest degradation efficiency of 49% was observed. While 5 mA cm^−2^ & 7 mA cm^−2^ gave 88% & 89% respectively. However, since current generated is related to energy consumption, the optimal current density of 5 mA cm^−2^ was used for this study. Moreover, the effect of current density was also investigated for the pristine BiOBr & Bi_2_O_2_S to show the effect of heterojunction formation. As shown in Fig. S10 for BiOBr, degradation efficiency of 37%, 49%, & 53% and for Bi_2_O_2_S (Fig. S11), degradation efficiency of 27%, 63% & 35% were observed for 2 mA cm^−2^, 5 mA cm^−2^ & 7 mA cm^−2^ respectively. This shows that BiOBr & Bi_2_O_2_S yield a lower degradation efficiency when compared with BiOBr/Bi_2_O_2_S. And at 7 mA cm^−2^, Bi_2_O_2_S electrode experienced leaching showing that it unstable after 60 min.

In addition, the efficiency of the BiOBr/Bi_2_O_2_S photoanode in different environments were investigated using CIP solution at different pH and two other organic pollutants (sulfamethoxazole and tetracycline). At pH 3, pH 7, and pH 10 of CIP solutions, degradation efficiencies of 71%, 88%, & 71% were recorded (Fig. 4c) where the pH of 7 gave the highest degradation efficiency. Ciprofloxacin has two p*K*_a_s values of 6.09 (carboxylic group is deprotonated) and 8.62 (the amino group is deprotonated).^55^ This implies that at pH 3 and pH 10, CIP will exist primarily in its cationic and anionic form respectively which might not be favourable due to electrostatic repulsion, thus in the zwitterionic form at pH 7, electrostatic reaction can be enhanced, thus leading to enhanced degradation efficiency. Also, the degradation efficiency of sulfamethoxazole (pH 6.7) and tetracycline (pH 7) gave 71% and 63% respectively (Fig. 4c). Although, the three analytes degraded in the similar pH but gave different results. This suggests that the simplicity of complexity of the structure of the analyte is one of the factors that can affect the degradation efficiency of a photoanode.

The total organic carbon (TOC) percentage removal of ciprofloxacin using BiOBr/Bi_2_O_2_S photoanode was investigated. As shown in Fig. S12, the initial TOC concentration was 30.5 ppm, and after degradation, the final TOC was 12.21 ppm. From eqn (6), the TOC removal was found to be 60%.6
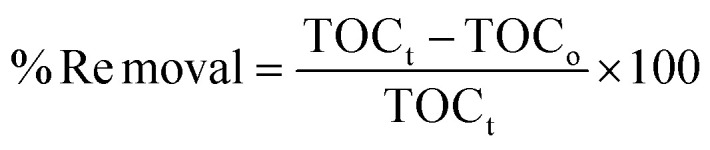
where TOC_t_ = final total organic carbon concentration (ppm), TOC = initial total organic carbon concentration (ppm).

Therefore, the heterojunction of BiOBr and Bi_2_O_2_S resulted in better photoelectrocatalytic activity, which is attributed to effective charge transfer and separation.

#### 3.5.2. Synergy and kinetics studies

**Fig. 5:**
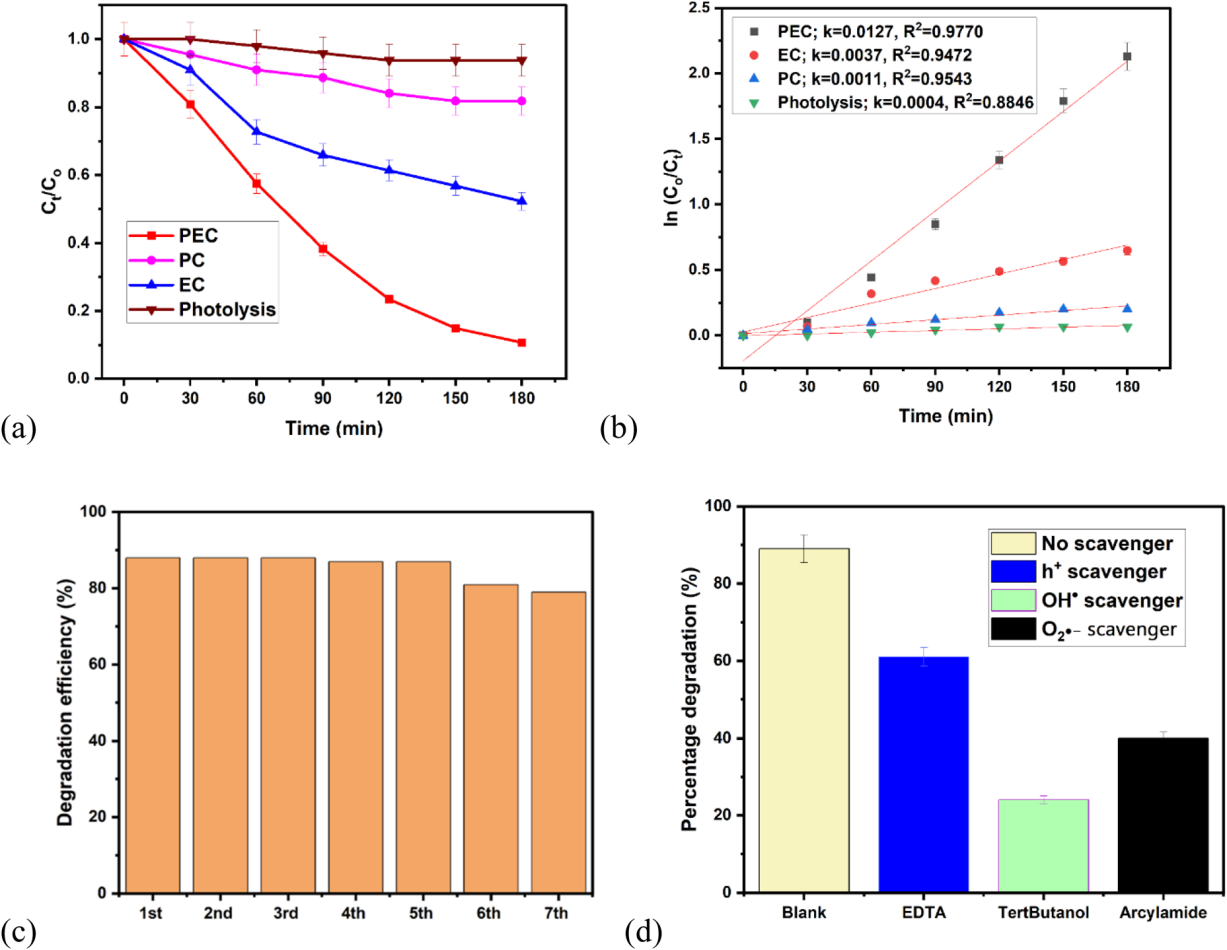
PEC degradation efficiency plot showing (a) synergistic effect, (b) kinetics study, (c) reusability study. (d) Scavenger study (experimental conditions: ciprofloxacin concentration = 5 mg L^−1^ in 0.1 M Na_2_SO_4_, current density = 5 mA cm^−2^, pH = 7).

The synergistic effect of the electrochemical oxidation (EC) and photocatalysis (PC) processes were investigated. PEC degradation of ciprofloxacin over BiOBr/Bi_2_O_2_S photoanode gave the highest degradation efficiency when compared to EC (48%), and PC (18%), and photolysis (16%) processes (Fig. 5a). This explains the synergistic effect of the electrochemical oxidation and photocatalysis processes involved in PEC degradation process are responsible for the enhanced PEC degradation efficiency. Therefore, PEC offers the most effective degradation route due to the combined effect of light and electrical energy which enhances reactive species generation. In addition, the data obtained were fitted linearly using the pseudo first order reaction model to obtain the rate of rection involved in these processes. As displayed in Fig. 5b, the *k* values obtained for PEC, EC, PC, and photolysis are 0.0127 min^−1^, 0.0037 min^−1^, 0.0011 min^−1^, and 0.004 min^−1^ respectively. This shows that the fastest rate of reaction was observed in PEC. Furthermore, the synergic factor (SF) of 1.44 was calculated from eqn (7).7
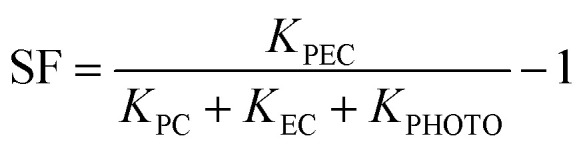


#### 3.5.3. Reusability experiment and radical determination

The stability and reusability of BiOBr/Bi_2_O_2_S photoanode were tested through a 7-cycle of degradation of ciprofloxacin (Fig. 5c). The difference of approximately 1% was observed in the first 5 cycles, then by the 6th cycle, the degradation efficiency had reduced from 88% to 81%. And, by the 7th cycle, the degradation efficiency reduced to 79%. Hence, a reduction of 9% was observed after 7-cycle treatment suggesting that the photoanode is stable. To further investigate the stability of the photoanode, the TEM analysis was conducted after the degradation process. As shown in Fig. S13, no observable changes were noted in the morphology as the observed nanorods and nanosheets were still intact. Furthermore, the BiOBr/Bi_2_O_2_S photoanode was subjected to XRD analysis after the degradation process. As shown in Fig. S14, all characteristic peaks of BiOBr/Bi_2_O_2_S remained visible. The additional peaks observed at approximately 28.8° and 36.4° correspond to polyvinylidene fluoride (PVDF)^56^ and fluorine-doped tin oxide (FTO),^57^ respectively, which were used in the electrode fabrication process.

Radical trapping experiment was used to investigate the reactive species that are most responsible for the degradation of ciprofloxacin over BiOBr/Bi_2_O_2_S photoanode. And, as revealed in Fig. 5d, the degradation efficiency reduced from 88% to 61% when EDTA (hole scavenger)^58^ was added to the solution, while scavenging of hydroxyl radical and superoxide anion radical gave 24% and 40% respectively. The scavenging of reactive species experiment shows that the primary reactive specie that contributed to the degradation of ciprofloxacin is the hydroxyl radical followed by the superoxide anion radical which played a secondary role. However, photogenerated hole also contributed to the degradation process.

### 3.6. Degradation product and pathways

**Scheme 1:**
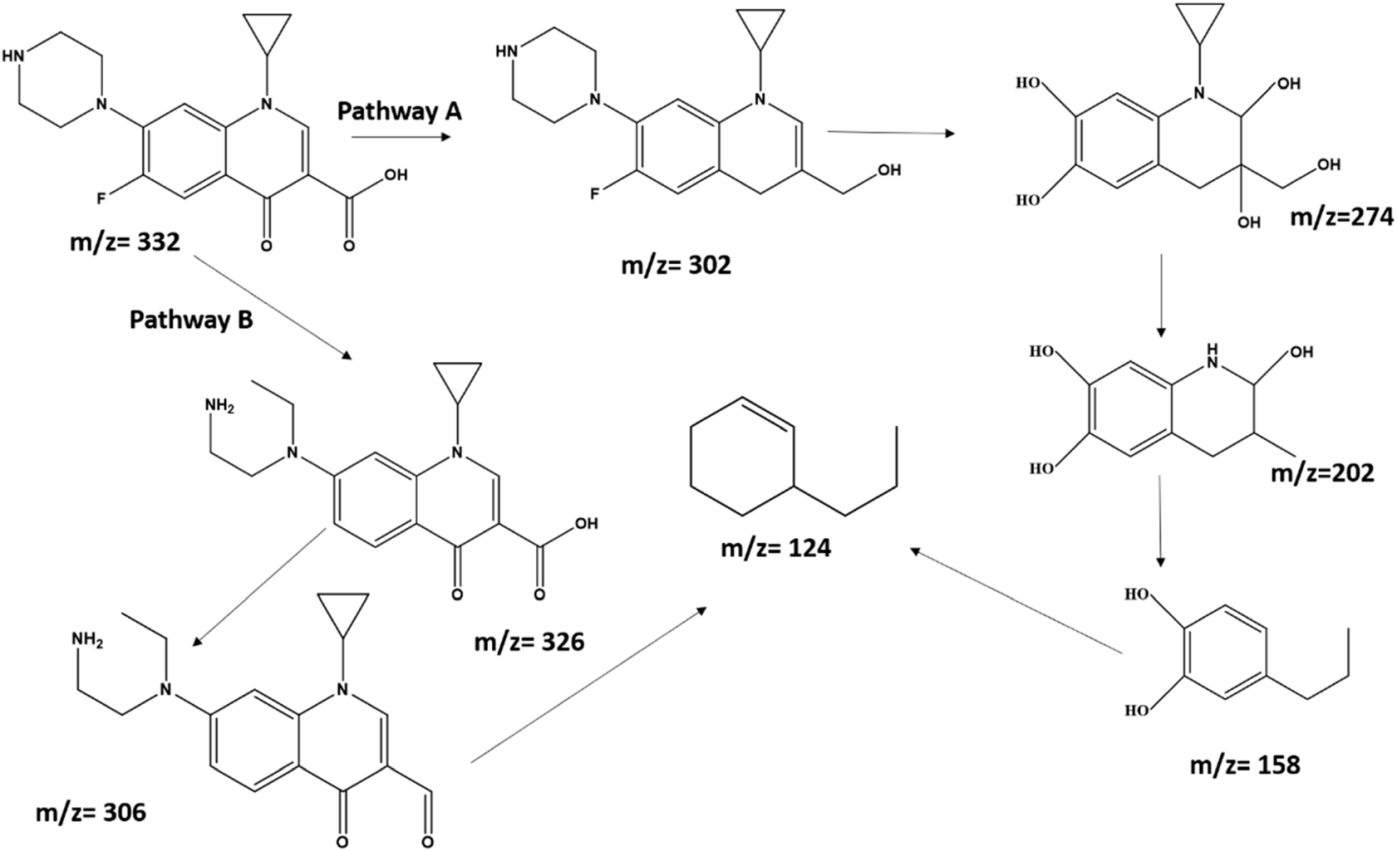
Proposed pathway and degradation products of ciprofloxacin.

The PEC oxidation of ciprofloxacin over BiOBr/Bi_2_O_2_S can lead to the formation of intermediates and by products. UPLC-MS was used to deduce the proposed degradation pathway and products formed. The degradation products were observed to have formed after 1 h degradation process (Fig. S15), with the parent peak of ciprofloxacin at *m*/*z* 332.14 almost disappearing after. As displayed in Scheme 1, two possible pathways were deduced from the mass to charge ratio (*m*/*z*) in the spectra. Pathway A suggests that steps involving hydroxyl radical initiated reaction led to dehydroxylation of carbonyl group in (*m*/*z* 302), then further oxidation led to the cleavage of piperazine ring and hydrolysis (*m*/*z* 274), followed by the cleavage of the cyclopropyl ring and dehydroxylation (*m*/*z* 202), deamination then led to pyridine ring opening, demethylation and dehydroxylation, thereby, further oxidative reaction led to the formation of the cycloalkene by-product (*m*/*z* 124). On the other hand, Pathway B suggests that due to direct hole-initiated oxidation, the piperazine ring in ciprofloxacin was opened, and rearrangement led to the intermediate formed at *m*/*z* 326, then decarboxylation led to the formation of *m*/*z* 306 intermediate, which undergo further oxidative cleavage to form the cycloalkene by-product (*m*/*z* 124). These intermediates and by-product are unstable, and further oxidation can lead to mineralisation to form H_2_O and CO_2_.

### 3.7. Proposed heterojunction formation and mechanism of degradation

**Scheme 2:**
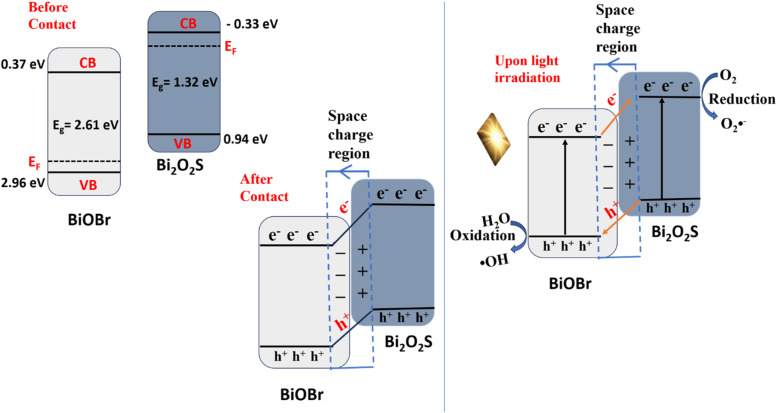
Proposed heterojunction formation of BiOBr/Bi_2_O_2_S and degradation mechanism.

The XPS valence analysis and band energy calculations of p-type BiOBr and n-type Bi_2_O_2_S show a typical type II band alignment in the heterojunction formation. Hence, the charge transfer mechanism in a type II heterojunction formation would suggest that the h^+^ in the VB of Bi_2_O_2_S and the e^−^ in the CB of BiOBr would take part in the oxidation and reduction processes, leading to the degradation of ciprofloxacin. However, the scavenger studies revealed that the hydroxyl radical and superoxide anion radical play a major role in the degradation of ciprofloxacin, with a minor contribution of photogenerated holes. Therefore, the type II heterojunction model cannot be used to explain the mechanism involved in BiOBr/Bi_2_O_2_S heterojunction, because the VB of Bi_2_O_2_S does not possess sufficient oxidation potential required to oxidise water to generate hydroxyl radical (˙OH/H_2_O = +2.27 V) and the CB of BiOBr does not possess sufficient reduction potential required to reduced absorbed oxygen to superoxide anion radical (O_2_/O_2_˙^−^ = −0.33 V). In addition, the result shows that the preserved photogenerated charge carriers with the highest oxidation and reduction ability were used in the degradation of ciprofloxacin. This makes the charge transfer mechanism involve resemble the *Z*-scheme heterojunction. However, the XPS analysis shows that the heterojunction formation in BiOBr/Bi_2_O_2_S was because of electron redistribution at the interface which led to the formation of space charge transfer which reveals the charge separation observed by the photoanode. With this evidence, we propose that a p–n junction was formed between the p-type BiOBr and n-type Bi_2_O_2_S photoanode and it is responsible for the mechanism involved in the reaction. Herein, as displayed in Scheme 2 upon contact between Bi_2_O_2_S and BiOBr, electrons will flow from Bi_2_O_2_S (electron-rich) to BiOBr, causing the surface of Bi_2_O_2_S to be positively charged, and photogenerated holes will migrate from BiOBr (hole rich) to Bi_2_O_2_S, causing the surface of BiOBr to be negatively charged. The redistribution of charges at the interface of BiOBr/Bi_2_O_2_S will lead to the formation of space charge region, thus leading to band bending and a built-in electric field, which creates a barrier that reduces the rate of recombination of electron–hole pairs. Upon light irradiation, the photogenerated holes will be drawn to the VB of BiOBr and the photogenerated electrons will be drawn to the CB of Bi_2_O_2_S where they participate in the pollutant removal process, and eqn (8)–(11) highlight the mechanism involved in the degradation process of ciprofloxacin. The heterojunction design combines the 2D structure of BiOBr and 1D structure of Bi_2_O_2_S to improve the interfacial contact area and for a p–n heterojunction which led to suppressed recombination rate and promotes the generation of reactive species for the degradation of organic pollutants in water.

8 BiOBr/Bi_2_O_2_S + *hν* → BiOBr/Bi_2_O_2_S (h^+^, e^−^)

9 H_2_O + h^+^ → H^+^ + ˙OH

10 e^−^ + O_2_ → O_2_˙^−^

11 ˙OH/O_2_˙^−^/h^+^ + CIP → smaller molecules → CO_2_ + H_2_O

## 4. Conclusion

A 2D/1D BiOBr/Bi_2_O_2_S heterojunction photoanode, with an optimised ratio of 20% Bi_2_O_2_S was used to degrade organic pollutants in water under visible light irradiation. The structural and electronic synergy, and charge redistribution within the heterojunction improve charge separation, visible light absorption, and generation of reactive species, leading to enhanced PEC degradation of the selected organic pollutants. The radical scavenging experiments highlight the primary role of hydroxyl radicals in the PEC degradation process of ciprofloxacin. The space charge region in the p–n heterojunction supports the proposed mechanism driven by the band alignment and charge redistribution, leading to suppressed recombination rate of the charge carriers. This work provides understanding into the optimisation and applicability of p–n heterojunction for broader environmental remediation applications.

## Author contributions

KD Jayeola: conceptualization, methodology, investigation, writing – original draft. DS Sipuka: methodology, writing – review & editing. TI Sebokolodi: methodology, writing – review & editing. JO Babalola: writing – review & editing, supervision. Y Zhao: writing – review & editing. OA Arotiba: methodology, writing – review & editing, supervision, funding acquisition, resources.

## Conflicts of interest

There are no conflicts of interest to declare.

## Supplementary Material

RA-015-D5RA03795F-s001

RA-015-D5RA03795F-s002

RA-015-D5RA03795F-s003

## Data Availability

The data supporting this article have been included as part of the SI. See DOI: https://doi.org/10.1039/d5ra03795f.
