# Supplementary material for: Redistribution of charge in a 2D/1D BiOBr/Bi2O2S heterojunction for the photoelectrocatalytic oxidation of organic pollutants in water
Source: RSC Adv. 2025 Aug 21;15(36):29680–91. doi: 10.1039/d5ra03795f (PMC12377251; doi:10.1039/d5ra03795f)
Supplement: RA-015-D5RA03795F-s001 [file RA-015-D5RA03795F-s001.pdf]

Supplementary Information (SI) for RSC Advances.
This journal is © The Royal Society of Chemistry 2025

SUPPORTING INFORMATION

Redistribution of charge in a 2D/1D BiOBr/Bi_2O_2S heterojunction for the photoelectrocatalytic oxidation of organic pollutants in water

Kehinde D Jayeola[a,b], Dimpo S Sipuka[a,b], Tsholofelo I. Sebokolodi[a,b], Jonathan O. Babalola[c,d], Yumeng Zhao[e], Omotayo A. Arotiba[a,b,*]

[a] Department of Chemical Sciences, University of Johannesburg, Doornfontein Johannesburg, South Africa

[b] Centre for Nanomaterials Science Research, University of Johannesburg, South Africa

[c] Department of Chemistry, University of Ibadan, Ibadan, Nigeria

[d] Bowen University, Iwo, Osun State, Nigeria

[e] State Key Laboratory of Urban Water Resource and Environment, Harbin Institute of Technology, Harbin 150090, China

Corresponding Author: oarotiba@uj.ac.za

Photoelectrochemical measurement and degradation

The photoelectrochemical measurement and degradation method used for this work has been reported in our previous work [1].

Photoelectrochemical measurements, including electrochemical impedance spectroscopy (EIS), photocurrent response, and Mott-Schottky measurements, were conducted using an Autolab PGSTAT204 potentiostat/galvanostat (Netherlands) employing a three-electrode setup. The fabricated electrodes served as the working electrode, while a platinum wire functioned as the counter electrode, and an Ag/AgCl (3 M KCl) electrode was employed as the reference electrode. The photoanode was exposed to irradiation from a 100 W solar simulator (Oriel LCA-100 model, USA), with a light density of 0.1 W/cm². For EIS, measurements were performed in a solution containing a 5 mM $[Fe(CN)_6]^{3/4-}$ in 0.1 M KCl with a +0.25 V vs. Ag/AgCl applied potential, covering a frequency range from 100 kHz to 0.1 Hz. Mott-Schottky measurements were recorded in the absence of light using a 5 mM $[Fe(CN)_6]^{3/4-}$ in 0.1 M KCl solution as the electrolyte. Photocurrent response data were evaluated under both dark and light conditions using a 0.1 M Na_2SO_4 solution, with an applied potential of 1.5 V vs. Ag/AgCl.

In a two- electrode system, the BiOBr/Bi_2O_2S photoanode as the working electrode and a platinum sheet was used as the cathode, the degradation experiments of the BiOBr/Bi_2O_2S photoanode were

conducted using a 50 mL quartz reaction cell containing 5 mg/L of sulfamethoxazole in 0.1 M Na_2SO_4. With an anode-cathode spacing of 3 cm, the photoanode was irradiated under a 100 W Xenon Lamp and the degradation efficiency of the pristine photoanodes and the composite photoanode were compared. The effects of pH and current density on the photoelectrochemical performance of the PEC process were examined. And, aliquots of the samples are collected at different time intervals using a disposable syringe and analysed using a UV-vis spectrophotometer.

The degradation efficiency of BiOBr/Bi_2O_2S photoanode was calculated using the formula Equation S1:

$$\text{Percentage degradation efficiency} = \frac{C_o - C_t}{C_o} * 100 \qquad \text{Eq. S1}$$

Where C_o= Initial concentration and C_t = Concentration at time t.

The percentage removal of total organic carbon was obtained with the formula in Equation S2

$$\text{Percentage extent of mineralisation (\%)} = \frac{TOC_o - TOC_t}{TOC_o} * 100 \qquad \text{Eq. S2}$$

Where TOC_o= Initial value before treatment and TOC_t = Final TOC after treatment at a particular time.

The main reactive species responsible for the degradation of ciprofloxacin over BiOBr/Bi_2O_2S photoanode via a free radical quenching experiment. Hydroxyl radicals, superoxide radicals, and photogenerated holes were trapped using 2 mM tert-butanol, 2 mM acrylamide, and 2 mM sodium ethylenediaminetetraacetate (EDTA) respectively.

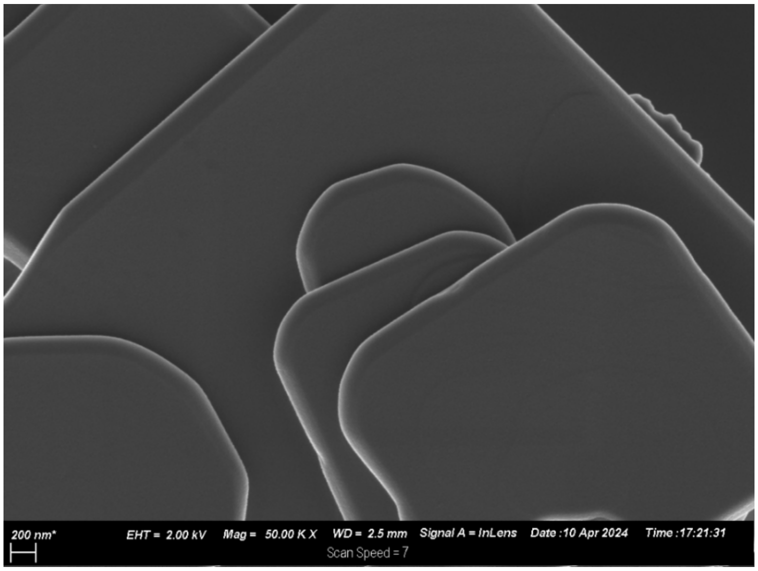

Figure S1: FESEM micrograph of BiOBr

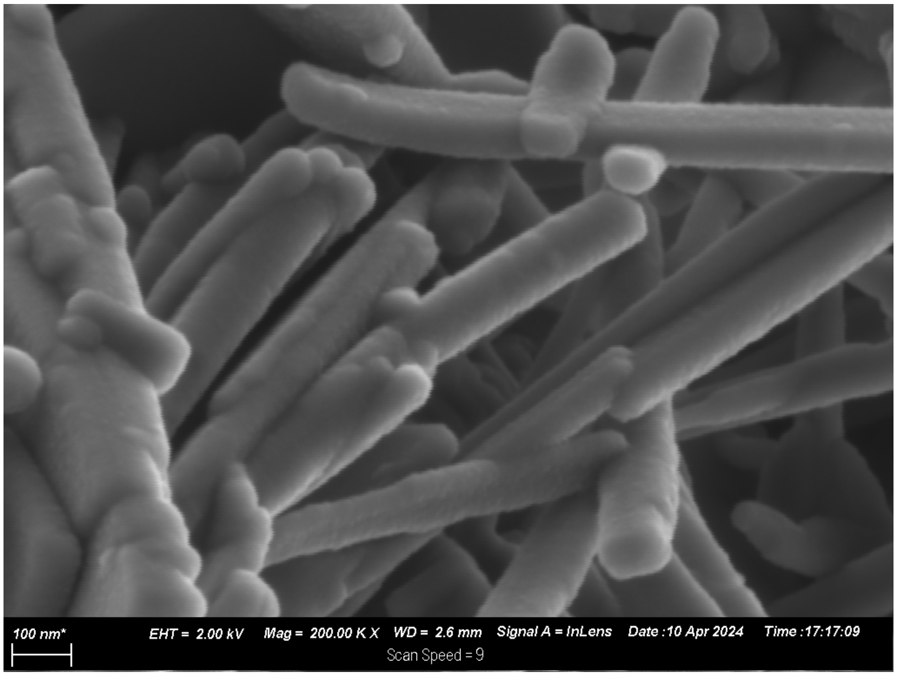

Figure S2: FESEM micrograph of Bi_2O_2S

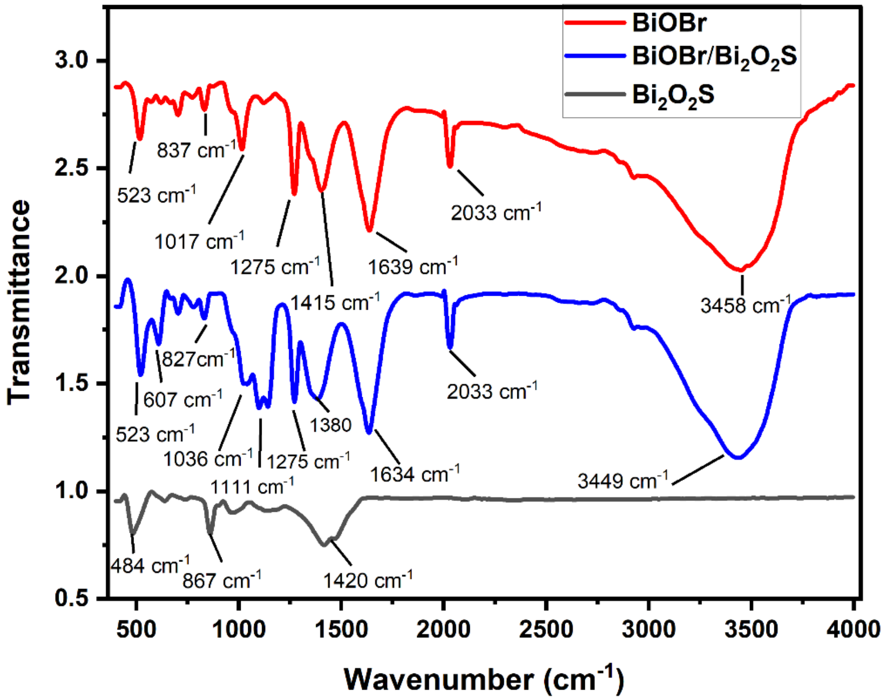

Figure S3: FTIR spectra of Bi_2O_2S, BiOBr, and BiOBr/ Bi_2O_2S

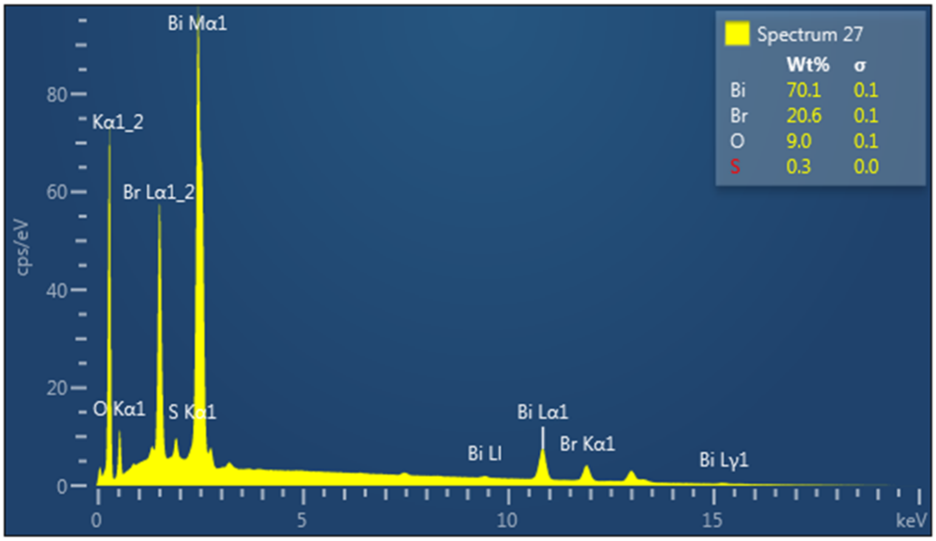

Figure S4: FESEM-EDX spectra of BiOBr/Bi_2O_2S

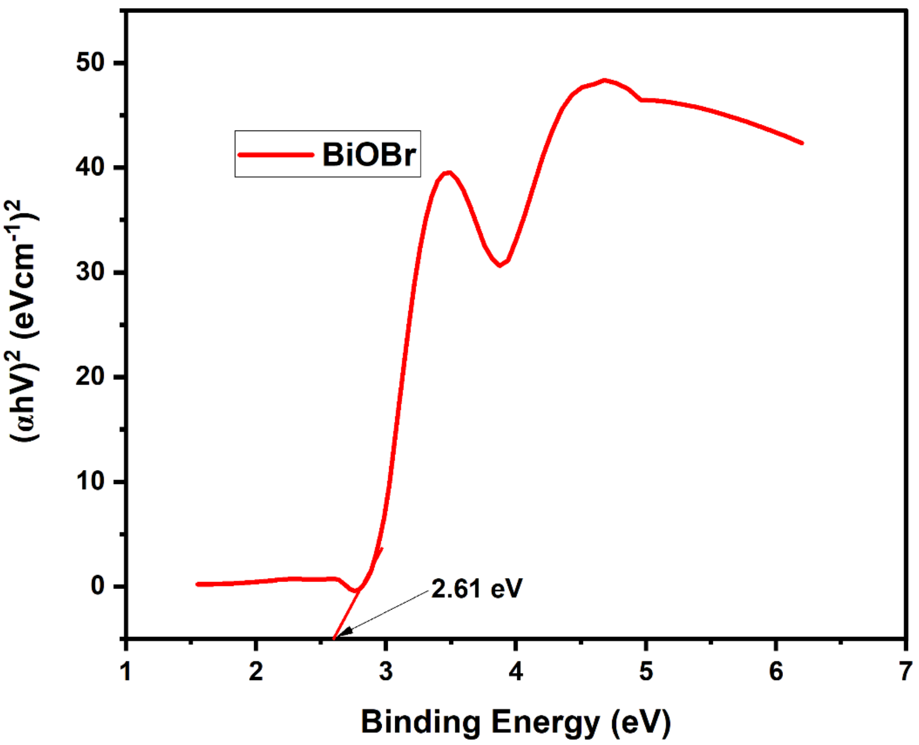

Figure S5: Tauc plot of BiOBr

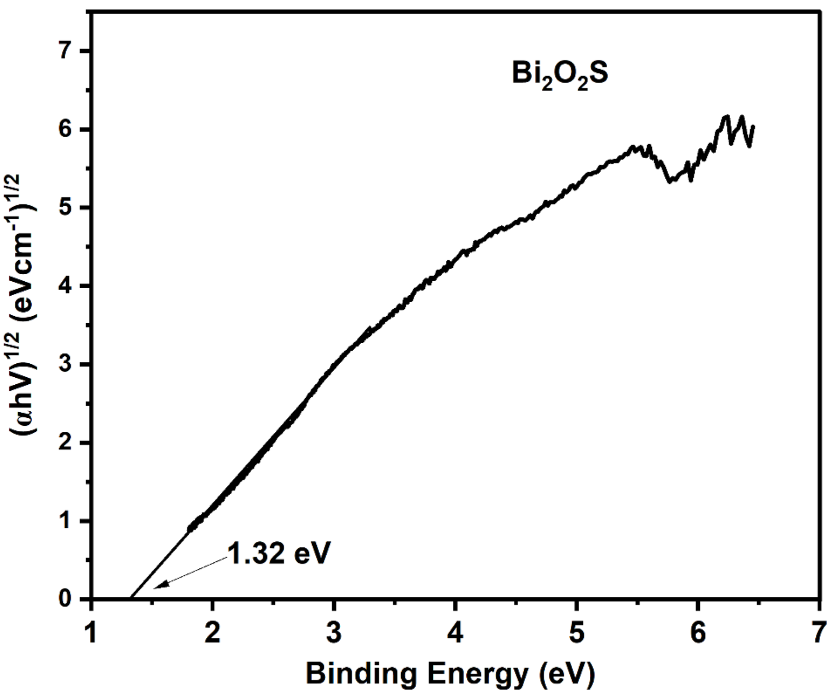

Figure S6: Tauc plot of Bi_2O_2S

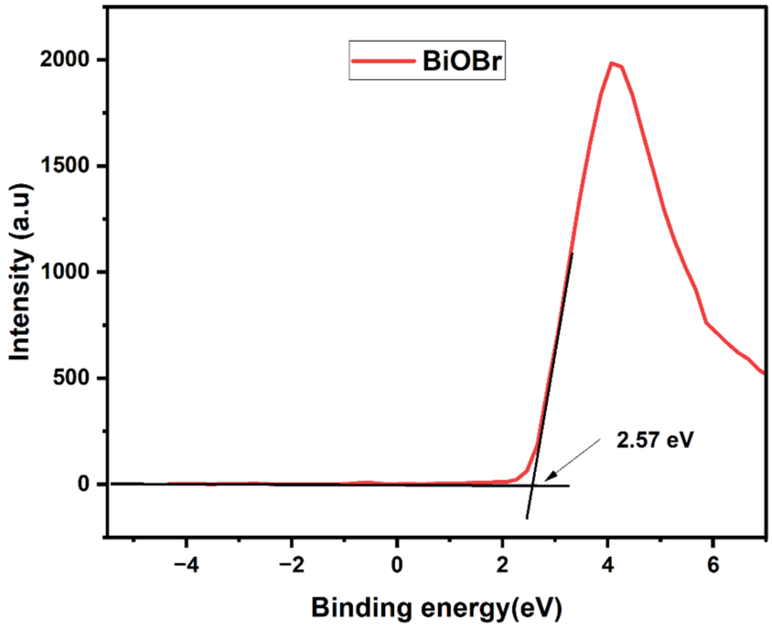

Figure S7: XPS valence spectra of BiOBr

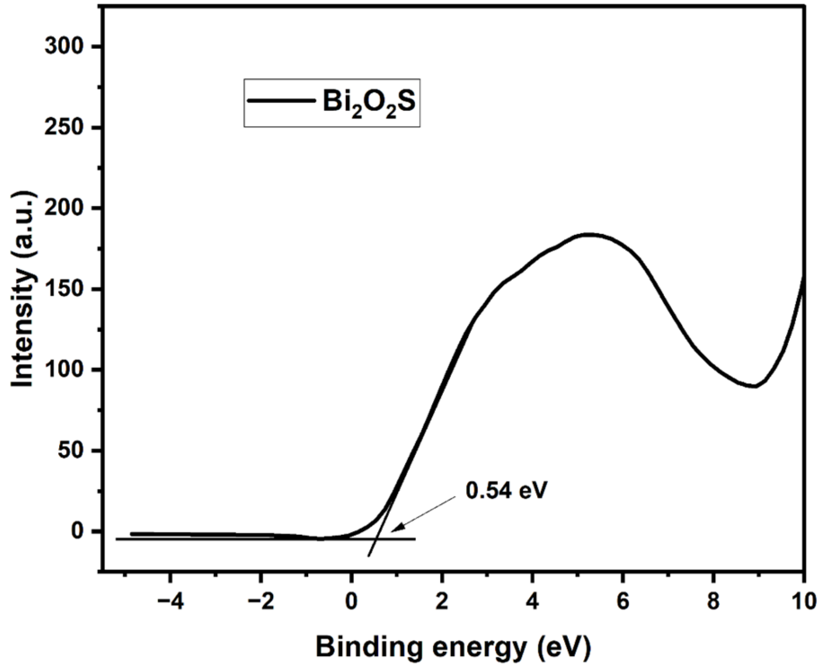

Figure S8: XPS valence spectra of Bi_2O_2S

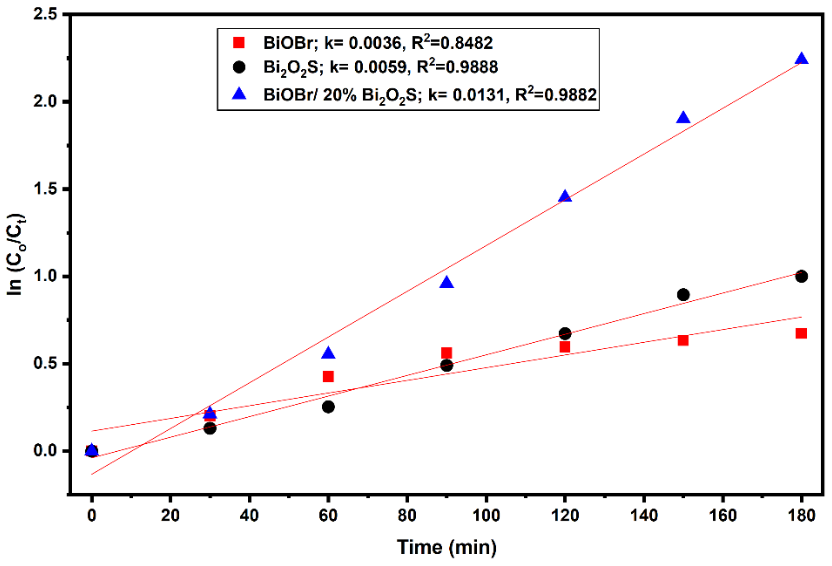

Figure S9: Kinetic studies of BiOBr, Bi_2O_2S & BiOBr/20% Bi_2O_2S for the degradation of Ciprofloxacin

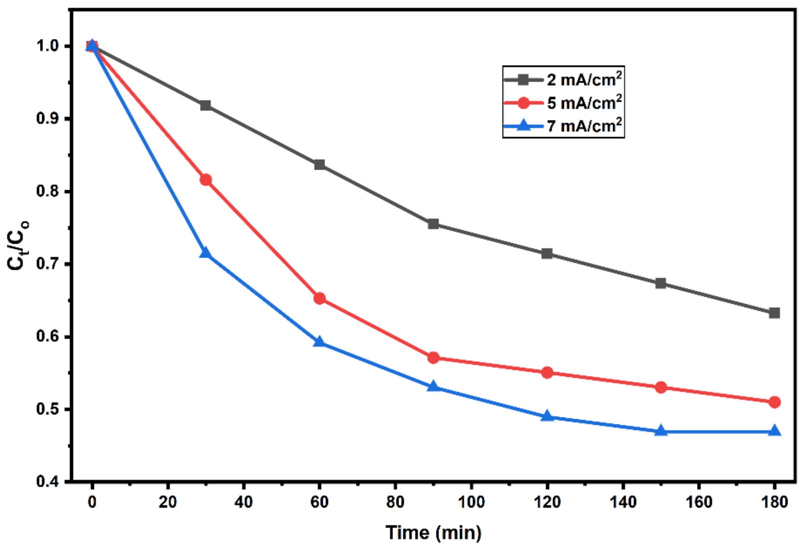

Figure S10: Effect of current density using BiOBr for the degradation of Ciprofloxacin

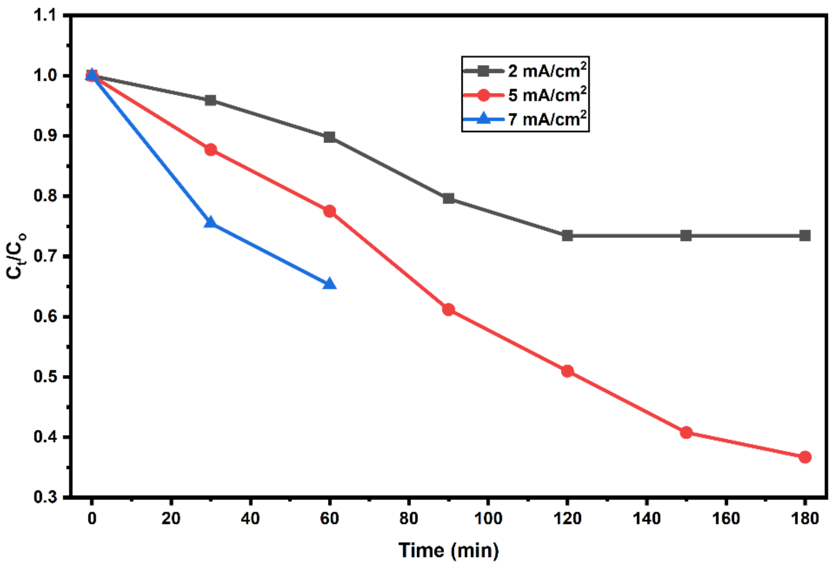

Figure S11: Effect of current density using Bi_2O_2S for the degradation of Ciprofloxacin

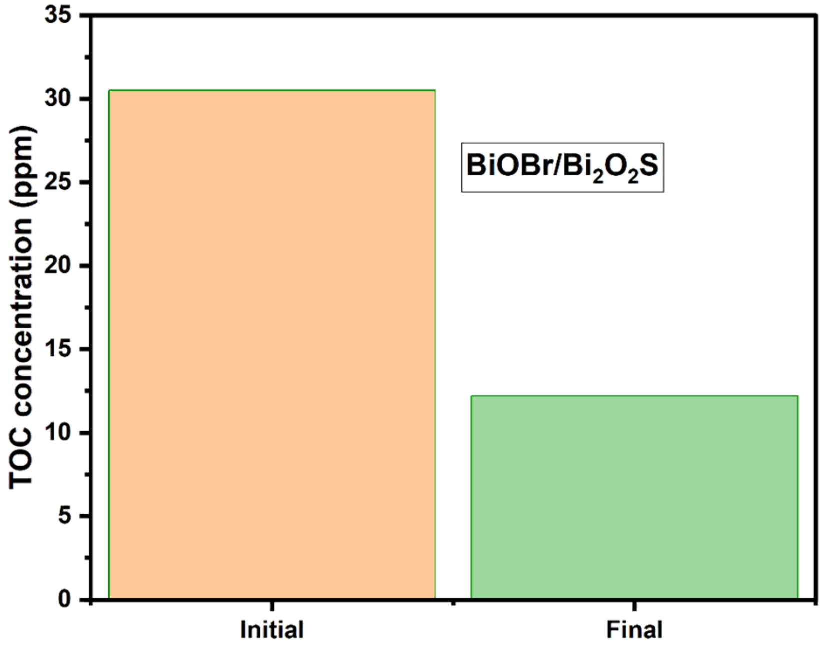

Figure S12: Total organic carbon removal of ciprofloxacin over $BiOBr/Bi_2O_2S$ photoanode

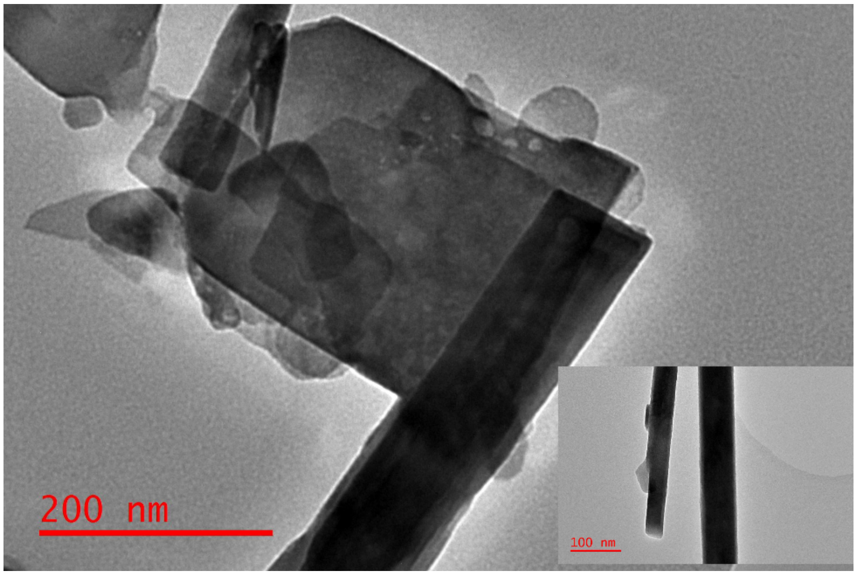

Figure S13: TEM micrograph of $BiOBr/Bi_2O_2S$ photoanode after degradation

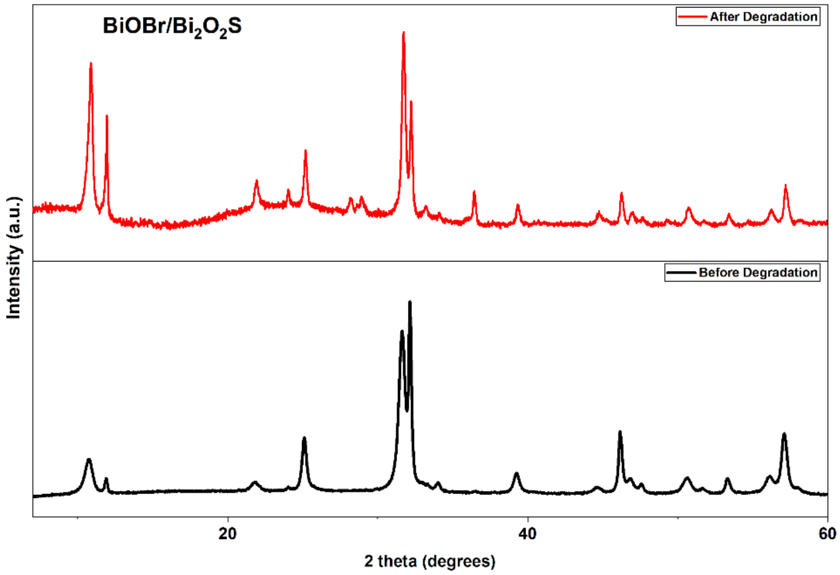

Figure S14: XRD analysis of $BiOBr/Bi_2O_2S$ photoanode after degradation

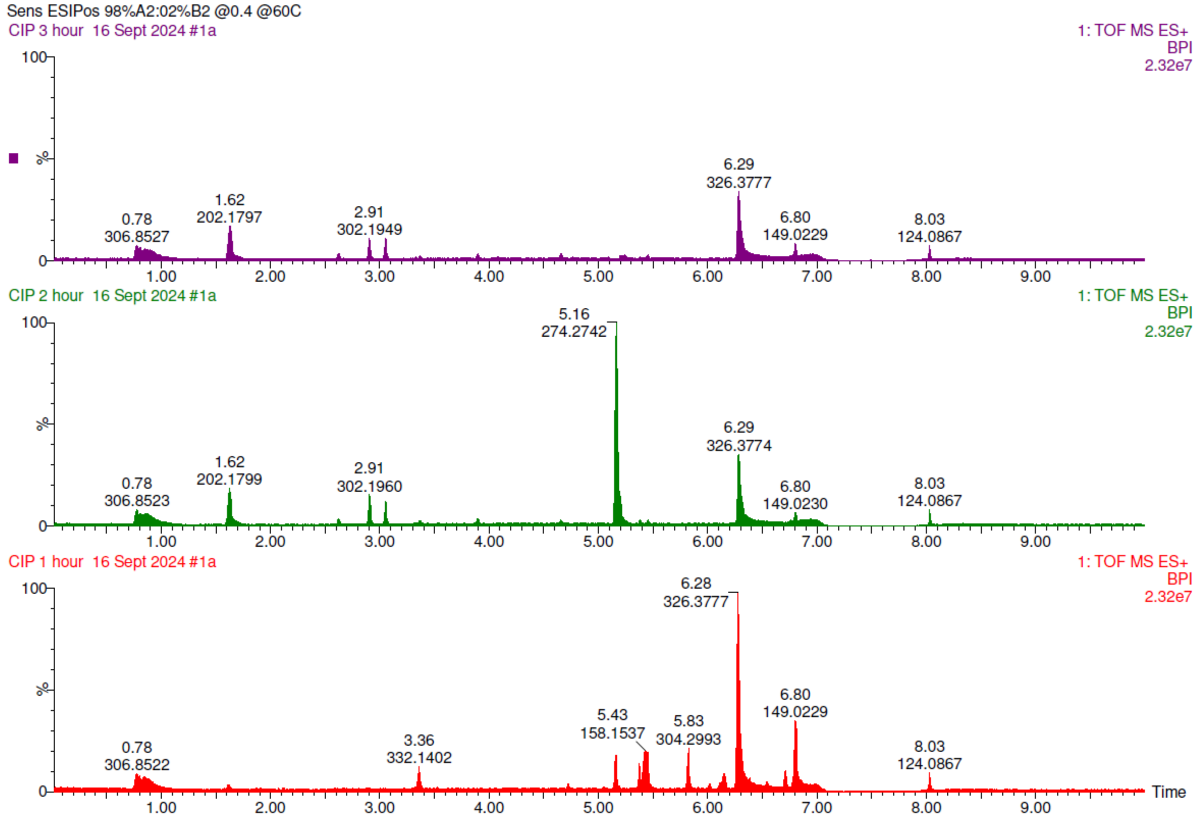

Figure S15: UPLC-MS spectra of the degradation of CIP over BiOBr/ Bi_2O_2S

References

1. K. D. Jayeola, D. S. Sipuka, T. I. Sebokolodi, O. V. Nkwachukwu, C. Muzenda, B. A. Koiki, J. O. Babalola, M. Zhou and O. A. Arotiba, *Chemical Engineering Journal*, 2024, **479**, 147482.
